# Where is the “g” in creativity? A specialization–differentiation hypothesis

**DOI:** 10.3389/fnhum.2014.01041

**Published:** 2015-01-13

**Authors:** Baptiste Barbot, Pablo P. L. Tinio

**Affiliations:** ^1^Individual Differences in Development Lab, Department of Psychology, Pace UniversityNew York, NY, USA; ^2^Child Study Center, Yale UniversityNew Haven, CT, USA; ^3^Department of Educational Foundations, Montclair State UniversityMontclair, NJ, USA

**Keywords:** creative potential, creative cognition, domain specificity, adolescent development, developmental neuroscience, puberty, motivated cognition

Unlike the construct of intelligence operationalized by the *g-factor*, there is limited evidence suggesting that creativity is a *domain-general* (i.e., as opposed to domain-specific) and a *generalized* (i.e., unitary) construct. However, there is a common and implicit *g-factor* view of creativity that potentially stems from the assumption that creativity represents an ability normally distributed in the human population (i.e., following a Gaussian distribution), ranging from everyday manifestations to eminent accomplishments. Indeed, individual differences exist in the outcome of human creative potential (i.e., particular combination of resources coming into play in creative work, including aspects of motivation, cognition, personality), but this does not suggest that creativity represents a *generalized* entity *per se*. This nuance is critical since many psychological and neuropsychological studies of creativity have made inferences about creativity as a generalized construct while relying on a set of highly specific tasks within highly specific domains, although there is limited support for the domain-generality of creativity. In fact, rare eminent individuals are those associated with exceptional creative achievement in multiple domains, and across multiple subdomains within a given field (Gray, 1966; Baer, 1998). The domain-specificity of creative behaviors and achievements is also repeatedly identified within general population samples (e.g., Carson et al., 2005; Silvia et al., 2009). In this article, we propose an alternative to the *g-factor* view of creativity: an organizing principle of the creative potential that involves its specialization through the formation of commitments and interests within a limited set of creative outlets. We outline evidence that such specialization arises during adolescence, a time during which biological maturational processes take place.

There is evidence that creativity simultaneously and *partially* involves: (1) a domain-general ability, (2) a set of domain-specific abilities, and (3) a set of task-relevant abilities (e.g., Lubart, 1999; Lubart and Guignard, 2004; Dietrich, 2007). This is due to the fact that the nature of creative work varies according to the creative domain, and even to the particular task constraints within that domain (e.g., Barbot and Lubart, 2012a). Correspondingly, several studies have shown that different modes of thinking involved in different types of creative work are accompanied by different patterns of brain activity (Razoumnikova, 2000; Fink et al., 2007; Sawyer, 2011; Kleibeuker et al., 2013b). A recent meta-analysis of 34 functional imaging studies of creative cognition has demonstrated the involvement of specific brain regions that align with general, domain-specific, and task-relevant aspects of the creative work (Gonen-Yaacovi et al., 2013). Not surprisingly, prefontal regions were associated with all creativity tasks investigated (representing the domain-general aspects or “common ground” across all tasks, or perhaps of all higher-cognitive functions; e.g., Dietrich, 2004; Reuter et al., 2005), while other regions were associated with distinct domain-specific areas (e.g., verbal vs. figural) and others with particular tasks (Gonen-Yaacovi et al., 2013). Showing high creative potential in a given task may, therefore, depend on the efficient recruitment of domain-general, domain-specific, and task-relevant brain regions.

Consistent with componential models, such efficient recruitment may reflect the confluence of multiple resources typically associated with creativity, including intelligence, knowledge, cognitive styles, personality, motivation, emotions, and aspects of the physical and socio-cultural contexts (e.g., Sternberg and Lubart, 1995). Based on this view, there is an established set of resources that seems to be involved in creative performance across domains and tasks (e.g., divergent thinking, openness to experiences, intrinsic motivation)—perhaps representing the domain-general aspects of creativity. To achieve a high level of creativity, an “optimal” combination of these resources is necessary, and such a combination could vary according to the domain or task under consideration (e.g., Barbot et al., 2011; Lubart et al., 2013). As an example, consider the set of skills involved in creative writing. Factors such as associative thinking and selective combination might be among the most important resources for writing poems, while perseverance and elaboration might be the most important resources for writing creative fiction (Barbot et al., 2012). Hence, due to the specific demands of these creative outlets, a different set of resources must come into play in a particular way to lead to creative outcomes. Together, all of these resources may be important for creative writing in general, along with a set of other domain-specific resources (e.g., vocabulary), and domain-general resources (e.g., divergent thinking, openness). According to this view, individuals have multiple potentials for being creative depending on the “fit” between their resources and the creative task demands (and contexts of time and place). For this reason, the probability of achieving an exceptional level of creativity in a given creative outlet is very low because a specific set of resources has to come into play in a specific way within the same person and at the right time and place. On the contrary, the vast majority of people will possess a combination of resources that does not optimally fit the demands of a specific creative outlet, time, and place, resulting in the majority of outcomes of “average” quality.

In sum, although some domain-general resources underlie creativity (Baer and Kaufman, 2005), they are less crucial than the interaction between person-level resources and the demands of the particular domain, task, time, and place. This lies in stark contrast to the view of creativity as a *generalized* trait or ability (Barbot et al., 2013b). Therefore, the quest for a *g-factor* of creativity might be unproductive. Rather, a more promising direction would be to focus on a central organizing principle that explains the optimal combination of all the person-level resources required in specific creative outlets. One such organizing principle is the formation of commitments within a limited set of creative domains or sub-domains that may lead to the specialization of a person's creative potential in these specific outlets. According to Plucker and Beghetto (2004), individuals themselves make their creativity a domain-specific entity by engaging in a field that interests them. Indeed, commitment to a creative task is an essential component of creative productivity, not only because it strengthens the motivational component of creativity, but also because as people invest in a specific domain, they have less time to devote to other areas. Therefore, the critical “source” of creativity may not be a *g-factor*, but instead the specialization of interests and commitments to a specific domain or creative outlet of interest, which in turn, facilitates the process of differentiation (development of a set of “specialized” skills needed for that particular outlet) of an individual's creative potential. This potential is differentiated because the individual has, voluntarily or not, specialized into a specific content area. With the emergence of domains of interest in which individuals focus their commitments, adolescence seems to be the critical time for the development of such “specialized” creative potential (Barbot and Lubart, 2012b).

This “specialization-differentiation” hypothesis is supported by studies that have emphasized the non-linearity of the development of creativity (e.g., Kleibeuker et al., 2013a), mirroring the non-normality of creative potential. Before the recent advancement of research on the neuroscience of creativity, scholars have outlined the critical role of puberty and associated biological processes for the development of creativity in its “adult form.” According to Albert (1996), there is a discontinuity between creative potential in childhood and in adolescence because each is associated with fairly distinct developmental conditions and pathways. Similarly, Rothenberg (1990) suggested that creativity in its adult form begins to develop as many biological and psychosocial changes transpire during adolescence. Neuroscientific evidence supports these suggestions. First, the adolescent brain is in a dynamic state and is characterized by tremendous neural plasticity (Blakemore and Choudhury, 2006). Because the prefrontal cortex represents the neural basis of higher cognitive functions including creative thinking (e.g., Dietrich, 2004), creativity develops in close relation with the thickness of this brain structure (e.g., Jung et al., 2010). At the onset of puberty and throughout adolescence, the prefrontal cortex is associated with a pruning process whereby neuronal connections that are used are strengthened, and those that are not are eliminated (Nelson and Guyer, 2011). This is evidenced by imaging studies showing a decrease of gray matter during adolescence (Raznahan et al., 2010), which in turn, might account for the creative cognition “slumps” that are often observed during adolescence (e.g., Charles and Runco, 2001). Occurring in parallel is the rapid decline in dopamine receptors at the onset of puberty (Teicher et al., 1995; Sisk and Foster, 2004). With fewer receptors to transmit signals, greater stimulation is required to activate the neurons, thus compelling adolescents to seek intense behavioral and emotional stimulations (Galván, 2010; Barbot and Hunter, 2012), behaviors that are themselves linked to aspects of personality that are important for creativity (e.g., sensation-seeking, risk-taking; Reuter et al., 2005). Together, these neurobiological dynamics are in line with studies that indicate a relative decrease of the cognitive aspects of creativity at the onset of puberty (particularly divergent thinking; e.g., Lau and Cheung, 2010), while the level of “divergent feeling” (including curiosity, complexity, risk-taking) increases dramatically (Claxton et al., 2005). Other neural processes salient in adolescence may contribute to specific aspects of the development and specialization of creative potential, notably the process of myelination associated with an enhanced integration of distributed brain areas (Spear, 2013), needed for the efficient recruitment of the regions associated with general, domain-specific, and task-relevant aspects of creativity. The *specialization–differentiation* hypothesis helps us understand contradictions in the sparse literature on the developmental aspects of creative potential in adolescence: individual differences in commitment to specific creative outlets may contribute to the discontinuous trajectories of creativity, as well as differences in the peak-age of creativity “slumps” and domains associated with such slumps.

Hence, it is possible that an adolescent committed to a specific creative outlet will strengthen the neurological substrates that underpin the resources critical for that particular outlet by “activating” them on a regular basis. Using the creative writing example, an adolescent who regularly creates poems will develop a set of task-relevant skills (e.g., associative thinking) that are associated with underlying neurological structures. Because writing poetry also “activates” a set of domain-specific skills (e.g., vocabulary), this adolescent will likely show creative potential not only in outlets that require a similar combination of task-relevant skills (e.g., various subtypes of poetry such as haiku or slam), but also, to some extent, in outlets that involve similar domain-specific skills (e.g., creative fiction). It is important to contrast this sort of *carryover* of the creative potential across *similar* creative tasks with the concept of domain-generality (reflecting a *g-factor* of creativity) for which we know there is limited evidence.

The *specialization–differentiation* hypothesis also finds support in several behavioral studies using training and transfer designs. They have shown that specific resources in one creativity domain can *partly* carryover in domains that share similar task-requirements (Baer, 1996; Barbot et al., 2013a; Onarheim and Friis-Olivarius, 2013). Developing multiple specialized skills needed to fit the requirements of various creative activities increases the odds of fitting the requirements of multiple tasks across domains, as long as these tasks share some of the features of the tasks in which the individual has specialized. Although this is not incompatible with the idea of a *Domain-General factor*, the contribution of such a factor may only be minimal.

In conclusion, the proposed *specialization–differentiation* hypothesis addresses current misconceptions of creativity as a domain-general and generalized entity. We propose that task-oriented commitment is an organizing principle of the creative potential. By specializing in creative domains and tasks of interest, a person's creative potential will progressively differentiate into task-relevant skills underlined by specific brain regions recruited on a regular basis. We posited that such process could peak during adolescence, given the neurobiological context associated with this period. Future longitudinal studies within the field of psychology and developmental neuroscience should explicitly test this hypothesis to uncover the developmental pathways leading to differentiated creative potentials in specific creativity outlets. To this end, work focusing on the neural systems underlying motivated behaviors (e.g., Ernst, 2014) as the basis of one's commitment to specific creative outlets may be particularly useful to test the mechanisms that eventually drive this organizing principle.

## Conflict of interest statement

The authors declare that the research was conducted in the absence of any commercial or financial relationships that could be construed as a potential conflict of interest.
